# Beyond synapses non-synaptic neural microenvironment interactions remodel circuits and drive glioma progression

**DOI:** 10.3389/fonc.2026.1793961

**Published:** 2026-05-13

**Authors:** Songyue Zhang, Helu Wang, Benlin Wang, Nan Chi, Qiaowei He, Yan Sun, Hongtao Zhang

**Affiliations:** 1 Faculty of Medicine, Macau University of Science and Technology, Macao, Macao SAR, China; 2 Neurosurgery Department of Yantai Yuhuangding Hospital, Yantai, China

**Keywords:** cancer neuroscience, glioma, neural microenvironment, neuromodulation, non-synaptic signaling, tumor microtubes

## Abstract

Genomic and epigenomic alterations alone cannot fully account for glioma infiltration, therapeutic resistance, or symptom severity. This review proposes a shift toward viewing the neural microenvironment as an active driver of glioma progression through diverse non-synaptic mechanisms, including metabolic coupling, ionic and volume transmission, gap junction signaling, and tumor microtube connectivity. We systematically map the contributions of astrocytes, oligodendrocyte precursor cell programs, microglia/macrophages, and the neurovascular unit, highlighting how their interactions contribute to local excitation-inhibition imbalances and disruptions in large-scale neural connectivity. These circuit-level disturbances closely correspond with clinically significant manifestations such as glioma-related epilepsy, cognitive deficits, and mood disorders, and also demonstrate correlations with patient survival outcomes. To rigorously connect molecular mechanisms to observable circuit disruptions, we integrate advanced methodologies including single-cell and spatial multi-omics analyses, human brain organoids and organotypic slice models, *in vivo* calcium imaging, and causal neuromodulation approaches. Emerging translational strategies identified by this approach include disrupting tumor microtube networks and gap junction-mediated signaling, functionally reprogramming glial cells, and employing targeted neuromodulation therapies. Additionally, we explore biomarker-driven combination therapies involving anti-angiogenic treatments and immunomodulatory agents as promising avenues for enhancing clinical outcomes.

## 1 Introduction

Gliomas are characterized by their invasive aggressiveness, recurrence, and heterogeneity. While there have been continual advances in imaging, surgical, and combined chemo- radiotherapy, there is still a poor prognosis overall. In the most recent WHO 2021 Classification of Central Nervous System Tumors (CNS5), molecular diagnostics are a main focus to classify and stratify tumors. However, variation in clinical phenotypes and outcomes has been shown to significantly exceed what traditional pathology is able to explain (1). Molecular advances include IDH mutation, 1p/19q co-deletion, and TERT promoter mutations, which significantly redefined the diagnostic landscape but were not sufficient to individually predict the symptom progression or functional prognosis of patients. More recent human studies have demonstrated that an increase in the functional integration of tumor cells with normal neural networks relates directly to poorer survival and poorer language function, demonstrating the network-level determinants (2–4). Additionally, the blood brain barrier/neurovascular unit (BBB/NVU) works to limit therapeutic delivery with drug therapies, but is also directly involved in invasion pathways and redirecting microenvironmental resources around tumors, ultimately making a strong case for a shift away from a cellular-genetic perspective to a more microenvironmental and neural-network focused perspective (5, 6).

In the past decade, the idea of “cancer neuroscience” has propelled this shift from correlation to causation. For example, optogenetic modulation of neuronal activity *in vivo* led to a direct increase in proliferation and growth of high-grade gliomas, confirming neuronal activity as a contributor to tumor progression (7, 8). Further studies used divergent optogenetic techniques to establish excitatory neuron-glioma synapse relationships/circumstances that showed AMPA receptor synaptic input resulted in direct influences impacting tumor proliferative signaling (9, 10). Concurrent studies have described tumor microtubes (TMs) connected via gap junctions creating electrical and metabolic networks allowing for long-distance invasion and resistance (11, 12). However, issues are not limited to synaptic domains. Tumors and astrocytes secrete glutamate using transporters such as cystine/glutamate antiporter system xc- (xCT) (SLC7A11), impeding clearance from excitatory amino acid transporters (EAAT). Together with gap junctions and ionic-volume transmission, this neurobiological disruption magnifies extrasynaptic excitation, and thereby enhances the invasive aspects of gliomas (13–15). Importantly, these networks can be therapeutically targeted through Connexin-43-related mechanisms of tumor invasiveness and chemoresistance and simply point to the consideration of non-synaptic approaches as new therapeutic entry points (16, 17).

Based on these considerations, this review will specifically examine the interactions occurring in the neural microenvironment outside of the synaptic paradigm. Our continued focus is on the ways that multiple cellular and structural components collectively remodel both local and cross-regional neural network signatures through metabolism, ionic balance, gap junctional coupling, and volumetric (ion) transmission. In addition, we will focus on how these alterations of the neural network are aligned with clinical symptoms of epilepsy, cognitive impairments, and emotional issues. As a method, single cell omics will help characterize cellular states and plasticity, while spatial transcriptomics will help clarify niche communication pathways. Complementing or combining human imaging and electrophysiology will allow comparability and human correlations to symptom definitions more objectively. Ultimately, we hope that this review will present elements of testable and translatable intervention strategies that strengthen our endeavours and ultimately broadened understanding of the inclusivity of neural networks beyond the singular synaptic-centered model.

## 2 Composition and functions of the neural microenvironment

### 2.1 Astrocytes: metabolic coupling, dysregulated glutamate clearance, and promotion of angiogenesis

Astrocytes are the principal cellular elements of the glioma microenvironment. Importantly, astrocytic endfeet cover the blood vessels at the neurovascular junction and aid in the maintenance of ionic balance during water homeostasis (18, 19). Endfeet cellular ion buffering mechanisms are dependent upon the membrane proteins’ inward-rectifying potassium channels (Kir4.1), as well as glutamate transporters that help keep extracellular K^+^ and glutamate under excitatory thresholds (20). Dysfunction of these membrane proteins leads to membrane depolarization and altering extracellular potassium/glutamate levels, augmenting the excitability within the network and enhancing the invasiveness of tumor cells (21, 22). The tumor environment may engage in active remodelling of the astrocytic-mediated endfeet structures and ion/water transport, facilitating invasion and highlighting a neuron-centric rather than tumor-only strategy to invade (23, 24). Astrocitic metabolism promotes the tumor with the action of lactate shuttle transporters (MCT1/4); connecting the actinicting process of metabolic buffering to ion and pH stabilization (25). The inhibition of metabolic coupling, with specific reference to MCT4, has potential therapeutic advantages for restricting tumor growth (26–28). Gliomas inherently have impaired astrocytic glutamate uptake through the EAAT2 transporter, compromising localistic glutamate accumulation to excessive extracellular levels (29). Rapid glutamate positioning elevates tumor-associated epilepsy and poorer prognosis in patients, postulating therapeutic modulation of xCT and EAAT as critical, dual approaches for anti-tumor and anti-epilepsy (30–33). Astrocytes actively remodel models of reactive lineages and transfers through gap-junctional coupling (Cx43) facilitating specifications of invasion and biological function of tumors, specifically by promoting signaling through VEGF (34–37).

### 2.2 Oligodendrocytes and oligodendrocyte precursor cells: coupling with tumor electrical outputs with nutritional network for stem-like properties

Oligodendrocyte precursor cells (OPCs) have a known extensive distribution within the Adult brain; displaying significant, electrophysiological activity (38). OPCs couple to neuronal network activity through AMPA receptor-mediated synaptic input through neurons (39, 40). In addition, OPCs can differentiate into functional oligodendrocytes, conduct formally within more local circuits and synaptic connections directly involving engulfment of presynaptic terminals to remap the local field (41, 42). Recent, single-cell multi-omic analysis of tumor host microenvironments engaged in tumor growth have identified a migratory “OPC-like” phenotype of malignant glioblastoma (GBM) cell types (43). Importantly, these GBM cell types have been shown to associate closely with networks of programs of tumor proliferation and aggression associated with invasion (44). Additionally, the GBM phenotype in part retains some canonic element of ascribed primary tissue, ex vivo myelin studies, and spheroid culture, suggesting that the “OPC-like” phenotype represents a conserved and coordinated cellular module based on tumor biology (45, 46). In a similar vein, human brain organoid models can exhibit into neural progenitor cell (NPC)/OPC states which can locally integrate into grow on the extent of local signalling in the microenvironment highlighting a model component of the OPC lineage might play in the tumor-host relationship (47, 48). Other tumor etiology, mice studies, can show that adult white matter OPCs can be induced to form glioma thought by pathway activation, specifically the biologically relevant platelet-derived growth factor (PDGF) pathway (49, 50). Most recently, molecular subclassification studies have determined a distinct proneural/OPC gene signature characterizes certain GBM subtypes while correlating with invasive behaviour (51). More broadly and contextually, active networks of OPCs receive synaptic inputs from both excitatory and inhibitory interneurons therefore providing the structural and functional cell basis for coupling to tumor-neuronal electrical networks (52, 53).

### 2.3 Microglia and tumor-associated macrophages: immune regulation, synaptic pruning, and promotion of tumor growth

Tumor-associated macrophages (TAMs) are the most common population in the non-tumor cellular compartment of GBM tissues (54). These include both resident brain microglia and peripheral monocyte-derived macrophages, and both are major contributors to the formation of a pro-invasive, angiogenic and immunosuppressive tumor microenvironment (55, 56). High-resolution single-cell studies have also demonstrated distinct immune landscapes across the different tumor types; for instance, primary gliomas are primarily composed of resident microglia, whereas brain metastases, as well as other cancers, exhibit significantly infiltrating peripheral immune cells, thereby indicating the tumor types own immune composition (57). In terms of therapeutics, blocking colony-stimulating factor 1 receptor (CSF-1R) signaling is not simply a process of depleting TAMs, but can also lead ultimately lead to re-programming the TAMs phenotypically, inhibit tumor progression, and potentially increase survival. This is an incredible finding, but also an indication of an immuno-microenvironmental targeting approach with substantial translational potential (58, 59). They also noted that ex vivo multinucleated microglia in human’s brain regulate neural network connectivity and excitation-inhibition balance through activity-dependent synaptic pruning via the activation of the complement cascade (C1q/C3) (60). Since glioma is a pro-inflammatory cancer, the inflammation induced by the tumor environment may exploit this pathway, and ultimately change the activity of circuits overseeing homeostasis and potentially elicit tumor-associated neurological symptoms in patients’ daily lives, as discussed earlier (61, 62). Trauma or IL-33s from tumor cells can further remodel immune-cell recruitment and activation to a pro-inflammatory-neural axis that promotes tumorigenesis and progression (63, 64).

### 2.4 Vasculature and blood-brain barrier: neurovascular unit as pathway for tumor invasion

**Figure 1:**
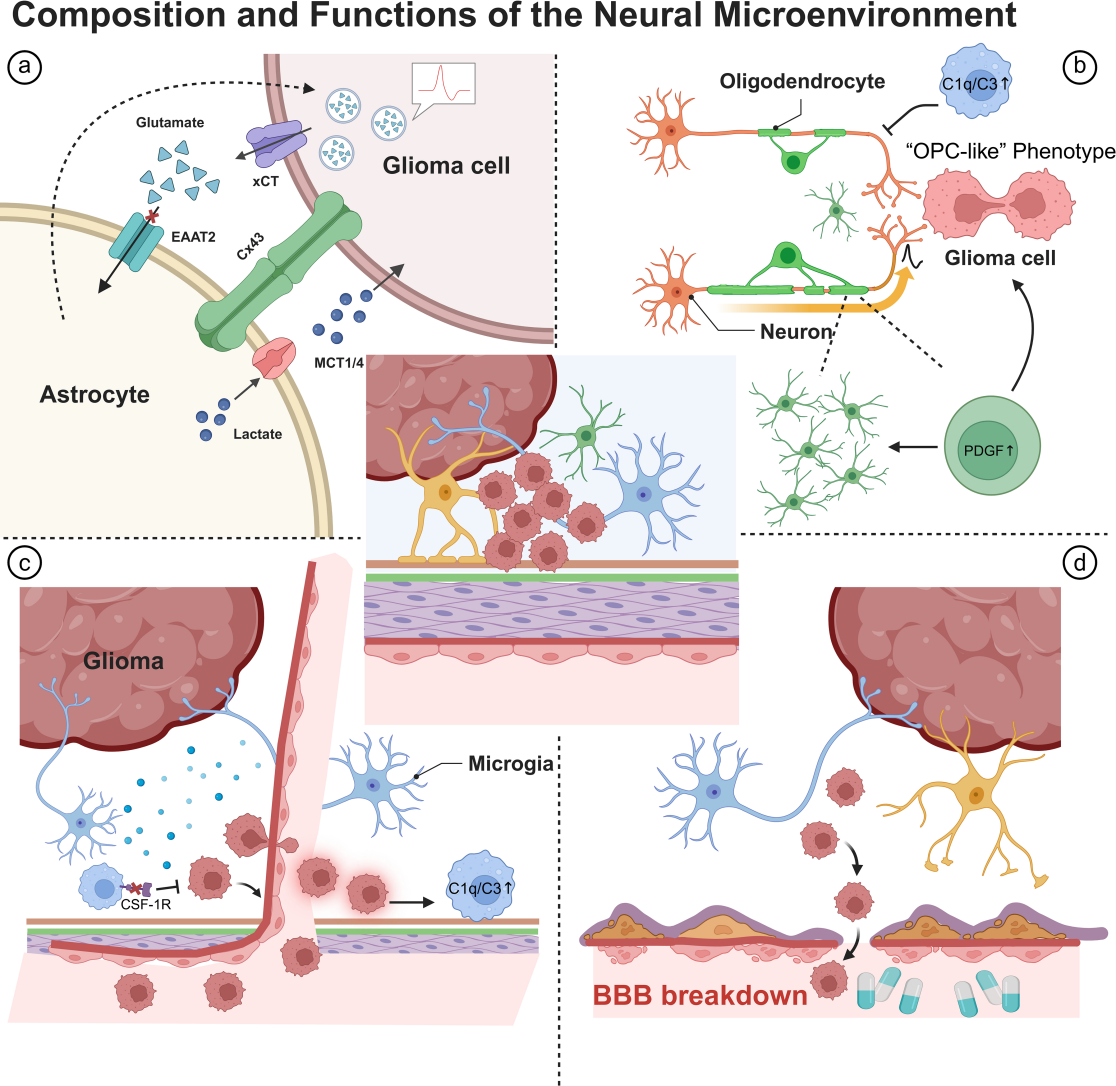
Neural microenvironment components and their non-synaptic roles in glioma. **(a)** Astrocytes: impaired EAAT2 with xCT-mediated glutamate release, Cx43 coupling, and MCT1/4 lactate shuttling. **(b)** Oligodendrocytes/OPCs: neuron–OPC synapses and PDGF-driven “OPC-like” tumor programs. **(c)** Microglia/macrophages: CSF-1R signaling and complement-mediated (C1q/C3) synaptic pruning. **(d)** Neurovascular unit: vascular co-option and BBB disruption enabling invasion.

Characterizing the blood-brain barrier (BBB) as an endothelial barrier is woefully inadequate since the BBB represents a multicellular and infiltrating assembly of targetable endothelial cells, pericytes, and astrocytic endfeet (65). Therefore, the physical integrity of the neurovascular unit actively modulates the homeostatic thresholds and molecular permeability of the relatively microenvironment (5, 66). In the first stages of invasion, glioma cells will initially hijack the variable blood supply using standard parental vascular co-option through locally affecting necessary focally disrupt BBB integrity, or ‘perivascular parasitism’, as alternative strategy of invasion independent of angiogenesis (6, 67). Furthermore, this vascular niche provides important perivascular signals and molecules to maintain self-renewal of glioma stem-like cells (GSCs) creating an integrated “vascular-stemness” axis for tumor progression (68, 69). Conversely, the drug transport and efflux mechanisms inherent to the BBB for size restrict therapeutic drug penetration and homogenous intratumoral global distribution to treat GBM presenting significant challenges. Innovative combined drug-delivery methods along with mechanisms of specific targeting to remodel the tumor microenvironment are essential to addressing these numerous challenges (70, 71). Together, these cellular and vascular interactions within the glioma microenvironment set the stage for alterations extending beyond individual cells, profoundly influencing the overall neural network function and activity patterns. See **Figure 1** for an overview of the neural microenvironment and its key non-synaptic interactions.

## 3 How gliomas remodel neural networks

### 3.1 Reprogramming neural activity: from local hyperexcitability to changes in synchronization

Diverse clinical and human *in vivo* studies originally offered evidence for a causal link within a network configuration, indicating that as functional connectivity increased between high-grade gliomas and surrounding non-pathologic brain tissue, gross survival time of patients with that disease modified as did language performance, demonstrating the network-level determinants (72–74). At the mechanistic level, gliomas induce cortical hyperexcitability and disorganization of electrical activity in neighboring cortical territories as a direct result of disrupted glutamate-ion homeostasis. Data from human-derived glioma models and translational reviews showed that pharmacological modulation or inhibition of system xCT reduces tumor-related glutamatergic drive and seizure burden, supporting a mechanism linking tumor-induced glutamate efflux and neuronal hyperexcitability (75–77).

Derived from imaging studies, glioma blood-oxygen-level-dependent (BOLD) responses showed correlated, synchronized oscillatory activity with distant brain regions, and the intensity of the oscillations correlated with patient survival indicating that tumor–brain network coupling is a quantifiable component of glioma severity (72, 78, 79). Utilizing intraoperative and/or perioperative electrocorticography (ECoG) 和 magnetoencephalography (MEG) recordings, it has been established that frequency-specific cortical disruptions and abnormal oscillatory activities could be identified within peritumoral cortex that also represent functional boundaries, which could be clinically useful to plan surgery, while also providing clinical monitoring with electrophysiological assessments (80,179).

### 3.2 TMs: the structural basis for tumor cell network development

Ultrastructurally, glioma cells connect with other glioma cells through long membranous extensions that form continuous networks. These TMs are traversing both white matter tracts, as well as cortical layers, and represent dual-function conduits involving invasion and electrical/metabolic communication (81–83). Tumor cells connected in networks demonstrate treatment-resistant and damage redistribution capabilities: the TMs are composed of gap junctions mediated by connexin 43 (Cx43) making it possible to propagate stress signals and engage in metabolic exchanges as cellular groups, permitting damage from local chemotherapy or radiation to be mitigated through collective cellular resilience (84). Once human and preclinical data confirmed that pharmacological modulation of Cx43/gap-junctional coupling can reduce electrical coupling and undermine collective resistance, it became possible to identify molecular targets for de-networking within the therapeutic framework (85). Tumor cell sub-populations embedded within the TM networks or the most connected or integrated in TMs exhibit the greatest therapeutic resistance and recurrence potential, which highlights the importance of developing selective therapeutic targets for these cells at the centre or network-integrated cells.

Beyond structural connectivity, TMs and gap junctions facilitate the functional propagation of intercellular calcium waves (ICWs), which are fundamental to glioma network communication. Importantly, this network is not functionally uniform but exhibits profound hierarchical heterogeneity. Recent studies have identified a specialized subpopulation of highly connected, intrinsically active cells functioning as network “pacemakers”. These pacemaker cells autonomously generate periodic calcium oscillations that propagate through the Cx43-coupled TM network. This rhythmic calcium signaling synchronizes the collective behavior of the tumor, actively driving cellular proliferation, coordinating invasion, and orchestrating collective resistance to cytotoxic therapies (82, 86). Consequently, calcium oscillations represent a targetable, active driver of glioma progression rather than a mere observational epiphenomenon.

### 3.3 Regulated by perisynaptic astrocytes: volume transmission

Perisynaptic astrocytes maintain the extracellular density of glutamate in the synaptic cleft low enough to prevent spillover activation of extrasynaptic receptors. When normal function is disrupted by the tumor, increased activation of extrasynaptic receptors, crosstalk between neighbouring synapses - involving increased excitability of neuronal networks-eventuates. Computational and experimental studies indicate the dynamics of transporters can be rapidly relayed to the distribution of extrasynaptic glutamate and neuronal excitability constitutes measurable intermediate mechanisms responsible for driving increased excitability through volume transmission (87). This literature aligns with epilepsy pathology associated with tumors, and supported with multimodal imaging/electrophysiological studies, with associated objective measures of relevance to classification and intervention in clinical contexts (88).

### 3.4 Circuit-level effects: local excitatory/inhibitory imbalance and cross-regional network reconfiguration

Studies in graph theory and connectomics show that gliomas generate functional perturbation in white matter and neural networks structures across the brain, beyond the lesion, and globally disrupt the small-world modularity of brain networks. Important brain networks exhibit abnormal connectivity patterns in glioma, suggesting a relation between these abnormal connectivity patterns and the functional impairment (89–91). Population-based findings indicate systemic disruption of network properties and vulnerability in brain areas away from the glioma. Furthermore, in glioma cases with distant brain tissue indicative of systemic compromise, these disrupted aspects of brain network properties correlate with survival as predictive factors, which implies that glioma should be viewed as a disorder of a brain network system (92). Language networks also exhibit the potential for complete hemispheric reorganization and prescription of new compensatory routes to illicit the recruitment of networks that can promote new compensatory processes. This is a key distinction to make when targeting a circuit therapeutically and further emphasizes the need for individualized care (93).

## 4 Neural microenvironment and clinical phenotypes

### 4.1 Epilepsy: circuit mechanisms of glioma associated epilepsy

The seizure phenomenon associated with brain tumors, termed brain tumor related epilepsy (BTRE), is produced through an increased state of network excitability. An increased state of network excitability is not simply localized, but instead is derived from modifications of the neural microenvironment due to primarily glutamate disturbance, but also due to disturbance in ionic homeostasis, inflamed processes, and modified vascular factors. Collectively these factors will lower the intrinsic firing threshold of the neurons and promote neuronal plasticity (94). Crucially, these microenvironmental shifts and the resulting network hyperexcitability are often initiated by specific upstream genetic aberrations within the tumor. For example, recent foundational studies demonstrate that glioma cells harboring PIK3CA variants can selectively secrete synapse-promoting factors that actively drive brain hyperactivity during early gliomagenesis. This establishes a direct causal link between the tumor’s distinct mutational landscape and the onset of macroscopic network excitability (94). The epileptogenic zone (EZ) that is generated from the mass of the tumor typically extends beyond the margin of the tumor and partially overlaps the infiltrative nature of the tumor. ECoG data that are collected during a awake craniotomy show certain spectral characteristic of spectral discharge will be distinctly characters of the tumor pathology (cortex) and adjacent areas of the cortex, which further establishes that the pathological electrical activity is the interface at the tumor-brain network (95). The evidence from the clinical electrophysiology and pathology suggests that removing the tumor and the ECoG cortical discharge has better functional outcomes than removing only the tumor. Moreover, current types of invasive surgery, such as stereoelectroencephalography (SEEG) and intraoperative cortical mapping likewise help with identifying applicable cortex that has been infiltrated by tumor and operational control of surgical margin with optimization of the parameters to achieve postoperative seizure control (96, 97). In practice, there is no current consensus guideline appearing in the international literature that endorses routine prophylactic intervention with antiepileptic drugs to all patients that are not experiencing seizures. Clinicians should not only determine the medication profile that is not effective with any chemotherapy, and identify the time for initiation and duration, but should also, apply individually timed perioperative and long-term seizure management strategies as guided by continued clinical monitoring with electrophysiological monitoring and neuroimaging (98).

### 4.2 Cognitive impairment: network remodeling and higher-order dysfunction

Individuals with high-grade gliomas often exhibit cognitive deficits in several domains, including attention, memory, language, and executive function. Individual symptomatology can vary from impairment of basic functions to distortion in functional network reorganization based on lesion location plus the reorganization of neural networks that can overlap across multiple regions, leading to measurable cognitive decline that is distant or away from the lesion area (99). Resting-state functional MRI has revealed systematic changes in the default mode network (DMN), including changes in the functional network’s connectivity strength, and offers insights into the emergence of deactivation patterns in the case of increased tumor burden or at advanced stages of treatment. These observable properties of the reorganization of the neural networks support a neural signature of cognitive symptoms (100). Parallel multimodal evaluations, in neuro-oncology, continue to provide evidence for the relevance of functional connectivity (FC) indices based on individual neuropsychological assessment scores, demonstrating potential utility for engaging these network indices as a clinical metric of susceptibility to cognitive impairment and continued monitoring of disease progression (101). In prognostication and patient stratification, resting-state patterns of the brain’s whole-connectome FC have characterized glioblastoma patients as short- or long-term survival phenotypes. Our results provide evidence for the adoption of networks as phenotypes in outcome models and tailored rehabilitative or neuromodulatory strategies (102). Beyond FC, connectomics is another level of prediction of preoperative cognitive performance in FC indices, which provide white matter connectivity patterns as an additional marker of potential cognitive outcomes, providing strong laboratory reports demonstrating that FC measures and cognitive outcomes offer direct correspondence in functional imaging data relevant for planning surgery, radiation, and other oncological treatments (103).

### 4.3 From reports of patients with psychiatric/emotion symptoms: interactions between the tumor and immune system, as well as neural circuits

**Figure 2:**
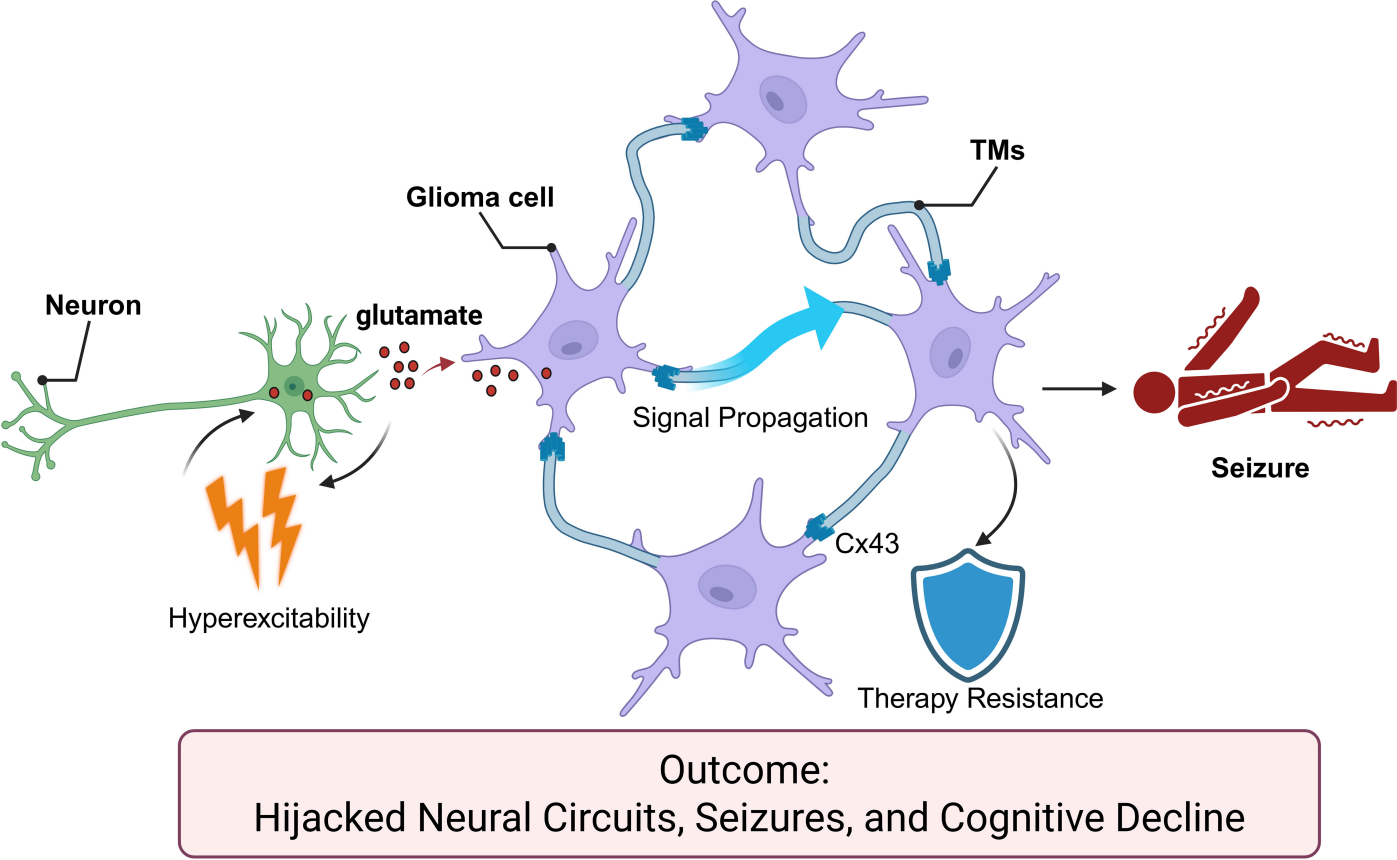
Non-synaptic tumor–glia–neuron coupling hijacks circuits. Glioma-released glutamate triggers neuronal hyperexcitability; tumor Cx43 gap junctions and tumor microtubes propagate signals and confer therapy resistance, culminating in seizures and cognitive decline.

The point prevalence rates of depression and anxiety in glioma patients are approximately 16% to 41% and 24% to 48%, respectively. Given that these disorders can have considerable correlations to patient quality of life and survival, their impact should be considered as a therapeutic issue and as a primary intervention, not secondary (104). Mechanistically, considerable oncology literature has reported that inflammatory pathways may relate to situational aspects of depressive symptoms, indicating that epithelial signals at a cancer-immune-brain interface can interact act on motivational and emotional circuits at neural-immune pathways (105). Therefore, anatomically and at the level of network, recent prospective cohort studies that tried to link symptoms of depression with tumor location and white matter disruption, have reported no consistent anatomical correspondence in a one-to-one manner at the group level, but that network mechanisms are more relevant than overall anatomy and pathology (106). Logically, there is overwhelming support for systematic reviews of clinical evidence or health services research recommending routine psychological screenings and access to psychosocial support, as well as including an integrated and symptom-oriented approach that is directed by cognitive and neural network attributes in neuro-oncological care (107). Given these diverse clinical manifestations and their underlying neural network mechanisms, the application of emerging research tools and advanced models becomes increasingly essential for dissecting complex glioma-host interactions, potentially enabling novel therapeutic approaches and personalized clinical interventions. See **Figure 2** for the circuit-to-symptom cascade.

## 5 Emerging research tools and models

### 5.1 Spatial transcriptomics and single-cell omics: revealing intercellular communication networks and tissue niches

Spatial transcriptomics (ST), which spatially links transcriptomic data to tissue, received Nature Methods News’ “Method of the Year” Award in 2020, reinforcing its maturity and applicability for the development of disease tissue atlases (108). For gliomas, multi-omic spatial transcriptomics helps reconstruct companies of cellular states with local immune and metabolic microenvironments while unpacking spatially segregated lineage states that exhibit adaptive rearrangements in the face of inflammatory or metabolic forces. This demonstrates a bidirectional dependence, or relativism, between tumor-intrinsic programs and the microenvironmental host (109). A number of more recent prospective cohorts have utilized single-nucleus or single-cell transcriptomics, chromatin accessibility, and ST/proteomic imaging on matched primary and recurrent samples, revealing stroma-like transitions in addition to shifts in immune composition during recurrence, to provide quantifiable trajectories predicting resistance of therapy and reoccurrence risk (110). However, ST faces inherent limitations, such as a trade-off between single-cell resolution and whole-transcriptome coverage, necessitating careful interpretation and computational deconvolution methods to accurately infer intercellular communication. Technically, ST still makes trade-offs between resolution and transcript coverage, and intercellular communication requires careful deconvolution regarding inference on ligand-receptor interactions. Recent reviews have provided comprehensive summaries about sources of bias and computational tools designed to facilitate better studies (111). Also, the ST platform’s original chip array supports reproducible, section-level whole-transcriptome profiling and has been developed as a shared platform for clinical and multi-center studies (112).

### 5.2 Organoid and brain slice co-culture models: humanized reconstruction of neuro–tumor interactions

Patient-derived glioblastoma organoids (GBOs) closely recapture the cellular heterogeneity, transcriptional landscapes, and morphology of primary tumors. They can be rapidly generated, cryopreserved, and are useful in drug sensitivity and immunotherapy testing, thereby embodying an important tool for integrated “patient–model–therapy” validation (113). Organoids can be co-cultured as tumor cells infiltrate the cerebral organoid along network-like projections and tissue structure to simulate the migratory behavior and microtubule connectivity observed in the human brain. GBOs are particularly suited as a high-throughput drug screening platform, coupled with quantitatively assessing the invasion dynamics of glioblastoma cells (114). Also, complementary organotypic brain slice models preserve myelin, vasculature, glial cells, and extracellular matrix which could provide a more physiologically-relevant microenvironment to study tumor invasion, drug response, and inflammatory networks, bridging *in vitro* and *in vivo* studies (115). However, organoids inherently lack complete vascularization and fully developed immune components, limiting their fidelity in replicating the full complexity of glioma biology. These limitations necessitate supplementary validation studies involving brain slices, co-cultures with endothelial or immune cells, and long-term dynamic imaging to improve physiological relevance. In translational studies, GBOs were combined with autologous CAR-T cells to test at the same time *in vivo* and *in vitro*. This approach generates rapid preclinical validation for immunotherapy dose/efficacy and tumor heterogeneity (116). Although organoids somewhat advance the field of cancer and multi-omic research, organoids are typically deficient in using vascular and immune elements or are not wholly mature. However, many of these issues can be addressed through validation studies using brain slices, co-culture with endothelial and immune cells, and long-term dynamic static imaging, thus improving understanding of non-synaptic cellular interactions (117).

### 5.3 *In vivo* imaging: capturing tumor–neural electrical coupling

The dynamic neural and tumor network activity can be gathered and spatially visualized using both *in vitro* and *in vivo* two-photon and calcium imaging techniques. *In vitro* calcium imaging, particularly when applied to patient-derived tumor models or organotypic brain slices, allows for the high-resolution mapping of network heterogeneity and the precise identification of localized pacemaker cell dynamics within a controlled microenvironment. Complementarily, *in vivo* methods are capable of capturing real-time neural activity and large-scale network interactions at millisecond-to-minute observations and micron-to-millimeter spatial resolutions. Those available imaging methodologies, along with their respective protocols will sit squarely at the intersection of tumor-neural coupling work (118). Specifically, *in vivo* two-photon imaging of glioma models has demonstrated time lapse imaging of tumor-associated macrophage sub-type behaviors on a spatio-temporal basis, allowing trajectory of their immune activity in coupling with brain networks and tumor invasion activity over time (119). Yet, these imaging techniques are constrained by limited penetration depth, potential phototoxicity, and high technical demands, which can restrict both temporal resolution and study duration. Moreover, accurate alignment between electrophysiological and imaging data requires careful calibration to avoid interpretational bias. Imaging and stimulations have developed to facilitate deep tissue imaging at millimeter-scaling fields, transitioning research methodologies from wide-field scanning to targeted imaging, along with the ability to scan longitudinally for tumor growth and microenvironmental occurrences (120). Dual-modality systems permit manipulation of neural tissue and cellular read-outs for the same living subject, and characterize causal relationships of an electrophysiological intervention response of tumor activity (121). Network measures collected *in vivo* can be mapped to imaging hotspots, providing parameter-driven guidance for organized surgical resections, radiotherapy planning, and symptom-based rehabilitation.

### 5.4 Neuromodulation, and human electrophysiology: establishing causal links with gold standard methods

Optogenetics provides sophisticated interrogation of neuronal activity, modulation of activity timing to the millisecond and specificity to cell type, connectivity, and projection identity. In the last decade, optogenetics has ‘cemented’ itself as the gold-standard for causal inference in circuit neuroscience. The following reference articles provide comprehensive reviews of optogenetics, the principles of delivery, its unifying theory, and its limitations of application (122). The gold-standard of causal inference has recently been ramped up to the human-scale. Neuropixels probes now provide high-density recordings of single-neuron activity inter-operatively, significantly reducing the translational distance of study from rodent and human models, and facilitating direct interrogation of disease circuitry across a multitude of neuro-physical conditions (123). Nonetheless, optogenetic and electrophysiological approaches are limited by their invasiveness, viral vector delivery risks, and ethical barriers in human applications. Furthermore, large-scale data acquisition demands specialized infrastructure and computational pipelines, which remain a challenge for routine translational studies. Closed-loop, wearable systems such as Neuro-stack, now enable single-unit and local field potential recordings across individuals free of magnetic constraints whilst providing stimulations, which give researchers the ability to couple the neural recordings, symptomatic behaviour, and stimulations parametrics in real-time, or near real-time (124). Research platforms that offer the same functionalities as CLoSES, enable signal extraction, decision making, and stimulation trigger in a near real-time pathway - thereby together with its developmental promises optimized and rapid translation from research to clinical context (125). In conclusion, the combination of spatial imaging, single-cell imaging, *in vivo* imaging, and stimulation/neuromodulation systems represents a closed loop pipeline, that can go from observational space, to locating the observation, enabling the manipulation of the identified observation, and furthermore validating. Provisions and operationalization will provide better and/or faster clinical utility by making spatially stratified sampling, rapid validation with GBOs in brain slices, human *in vivo* network biomarkers, and closed-loop stimulation protocols clinically relevant for translationally mapping non-synaptic cellular-relationships into systematic, parameterized measurable, controllable, falsifiable therapeutic strategies. By leveraging these advanced research methodologies and integrative models, we are now better equipped to translate our deepened understanding of glioma-neural network interactions into practical therapeutic strategies, which will be further discussed in the following section.

## 6 Therapeutic implications

An overview of these emerging therapeutic strategies, spanning from preclinical models to active clinical trials, is summarized in **Table 1**.

**Table 1:** Summary of non-synaptic and network-targeting therapeutic strategies in glioma.

Therapeutic target	Agent/modality	Mechanism of action	Developmental stage
Gap Junctions & TMs	Meclofenamate (MFA)	Disrupts Cx43 gap junctions and TMs, compromises cytosolic traffic, sensitizes to chemotherapy	Phase I/II Clinical Trial (MecMeth/NOA-24)
Gap Junctions & TMs	Tonabersat	Disrupts Cx43 gap junctions, enhances TMZ cytotoxicity, excellent BBB penetrance	Preclinical
Glutamate & Metabolism	Sulfasalazine (SSZ)	Inhibits xCT (system xc-), reduces glutamate efflux, induces ferroptosis	Clinical context (often limited by toxicity)
Glutamate & Metabolism	Lomitapide	Amplifies ROS and depletes CoQ10, sensitizes TMZ-resistant cells to ferroptosis	Preclinical
Glutamate Receptors	Perampanel	AMPA receptor antagonist, reduces peritumoral hyperexcitability and seizure burden	Clinical/Perioperative
Neuromodulation	Responsive Neurostimulation (RNS)	Closed-loop stimulation to restore excitatory-inhibitory balance and control seizures	Clinical (Brain tumor-related epilepsy)
Neuromodulation	rTMS & tDCS	Non-invasive modulation of structural plasticity for motor recovery and cognitive/language rehabilitation	Clinical (Postoperative/RCTs emerging)
Physical Barrier & Delivery	LIPU (SonoCloud-9)	Ultrasound-mediated BBB disruption to enhance widespread delivery of chemotherapeutics (e.g., carboplatin)	Phase I/II Clinical Trial
Physical Energy	Tumor Treating Fields (TTFields)	Alternating electric fields that disrupt TM integrity and mitotic processes	Clinical (Standard of care/active trials)
Immunotherapy	Locoregional CAR-T (e.g., IL-13Rα2)	Intraventricular delivery aligned with the infiltrative tumor network to overcome immunosuppression	Phase I Clinical Trials

### 6.1 Interfering with tumor electrical networks: targeting TMs and gap junctions

The “de-networking” strategy arises from evidence that gliomas deliberately integrated into larger, collective networks through TMs and gap junctions. This switch to systemic properties, such as invasiveness, bypassing injury, and later therapeutic resistance, suggests that attacking the structural and communicating nodes within this collective network may produce more effective and efficient results than simply killing one or more single cells (126). From a pharmacologic standpoint, meclofenamate (MFA), a gap junction inhibitor that can cross the blood-brain barrier, can compromise TM structures, disrupt cytoplasmic intercellular sharing, and importantly, sensitize preclinical models to alkylating agents. Therefore, it is feasible to pursue the gap junction–TMs axis as a therapeutic target of synthetic lethality (127). Clinical correlations are now underway with the multicenter MecMeth/NOA-24 trial, which is studying the safety and early efficacy of MFA and TMZ in recurrent GBM, which is a direct translational evolution from preclinical studies proving that resistance by TMs could be sensitized by inhibiting gap junctions (127). This trial employs a two-phase design, with Phase I focused on dose-escalation to assess dose-limiting toxicities within 8 weeks of combined MFA and TMZ treatment, and Phase II using a randomized parallel-group design evaluating progression-free survival as the primary outcome, along with overall survival, safety, KPS, and quality of life as secondary endpoints (128). TMZ and RT may each be aligned with potential predispositions for resistance, and therefore, route to convergence itself may signal benefit. In addition to meclofenamate, identifying clinically viable alternatives is crucial for expanding the de-networking therapeutic arsenal. For instance, the clinically validated gap junction modulator tonabersat has recently emerged as a highly promising alternative. Preclinical studies demonstrate that tonabersat effectively disrupts glioma cell connectivity and significantly enhances the cytotoxicity of TMZ. Crucially, compared to older generation inhibitors, tonabersat offers superior oral bioavailability and excellent blood-brain barrier (BBB) penetrance, positioning it as a highly translatable agent for dismantling glioma networks (129). Also, from our discussion earlier, the causal relationship in the Cx43–PI3Kβ signaling axis driving TMZ resistance lends credence to the potential of combined inhibition of gap junctions with inhibition to single activated pathway inhibitors in pursuit of overcoming a single-agent ceiling for therapeutic success (81).

Beyond the traditional approach of inhibiting gap junctions, recent studies have demonstrated that non-junctional Cx43 directly interacts with microtubules in glioblastoma stem-like cells, facilitating cell migration and invasion. This finding indicates that targeting the “Cx43–microtubule–microtubule bundle/tumor microtube (TMs)” axis itself could serve as a novel therapeutic strategy for disrupting tumor cell networks. Specifically, blocking this interaction can compromise the network integrity and mechanical migration capacity of tumor cells, thus laying the foundation for synthetic lethality when combined with chemotherapy or radiotherapy (130). It is noteworthy that the coupling of Cx43 with the cytoskeleton is not restricted to gap junction membrane domains; cytoplasmic Cx43 anchored to microtubules also maintains the stability required for collective cell migration. Consequently, future drug development strategies should carefully distinguish between gap junction channel functions and hemichannel or cytoplasmic-associated roles of Cx43. Such specificity would allow selective disruption of malignant cell networks without inducing additional adverse effects on neuronal networks (131).

### 6.2 Modulating glial cells: targeting astrocytes and OPCs to limit tumor spread

Astrocytes are cells that are mainly responsible for regulating the homeostasis of extracellular glutamate and ions, which directly impact excitability outside synapses. Therapeutic attempts to restore glutamate homeostasis are behind either the anti-tumor or anti-seizure, or both conditional effects of interventions available (132). The xCT pathway represents glutamate export and antioxidant properties in GBM, and through this mechanism, inhibition would induce ferroptosis and limit tumor invasion. That said, the clinical pairings of treatments such as SSZ with TMZ and RT have toxicity barriers which must be overcome with different dosing, schedules, and selection of patients (133–135). PDGFRA-directed approaches geared towards OPC-like programs have struggled even with monotherapy dasatinib. Similarly, while studies have preclinically combined dasatinib with mTOR inhibition (everolimus), dasatinib, or started with potent PDGFRA inhibitors with planning to show, coordinated multi-dimensional treatments exploring cell lineages thinking, modulating pathways that one believe contributes to GBM pathophysiology, and using drugs compatible with permeating the CNS, may be more effective (136, 137).

Within the “astrocyte–metabolism–excitotoxicity” axis, the MCT4/CD147 complex has been demonstrated to promote GBM proliferation, migration, and VEGF signaling. Inhibition of this complex reduces angiogenesis and tumor invasiveness in both *in vitro* and *in vivo* models, thereby providing a metabolic target for implementing a non-synaptic therapeutic strategy aimed at “inhibiting acid metabolism, stabilizing extracellular pH, and reducing network hyperexcitability” (138). Simultaneously, ferroptosis has emerged as a complementary pathway associated with the xCT/GSH axis. Preclinical translational research from 2025 indicated that lomitapide, through CoQ10 depletion and ROS amplification, sensitizes TMZ-resistant cells to ferroptosis. This finding suggests the potential to concurrently integrate “metabolism–stress–ferroptosis” and “anti-excitotoxicity” pathways within a unified therapeutic framework (139). At the neurovascular unit level, pericytes have been identified to enhance astrocytic EAAT-mediated glutamate uptake, providing microenvironmental evidence for restoring excitatory homeostasis locally. This finding aligns with our comprehensive concept in Chapter 6, emphasizing microenvironmental repolarization rather than mere cytotoxic killing strategies (140). Furthermore, a broad cancer review in 2024 explicitly emphasized the potential synergistic relationship between ferroptosis and immunotherapy/radiotherapy, supporting an integrated therapeutic exploration of symptom management (epilepsy control), neuronal network stability, and metabolic modulation, centered around xCT inhibition combined with radiotherapy and/or immunotherapy (141).

### 6.3 Electrophysiological approaches: neuromodulation for tumor treatment and symptom management

Distinctly involving neuromodulation to influence the structural plasticity of a neural network and, simultaneously, affect symptomatic plasticity is novel in neuro-oncology. For example, in glioma patients, navigated repetitive transcranial magnetic stimulation (rTMS) influenced motor recovery in the acute postoperative period. In defining further that intervening at the network level was both feasible and safe (142). RNS also offered reductions in seizure frequency, and improved quality of life, in cases of refractory tumor-associated epilepsy, which illustrated the viability of performing actual individual interventions in the electrophysiological realm (143). Involved in the idea of “excitability” extending tumor progression related to glioma, AMPA receptor blockade, for example with perampanel, has been considered perioperatively viable, which lends credence to combining pharmacological excitability modulation with neuromodulation (144).

Beyond case reports on responsive neurostimulation (RNS) and postoperative rTMS, randomized controlled trial evidence supporting targeted transcranial direct current stimulation (tDCS) of the language network is now beginning to emerge. A systematic study aimed at neuro-oncology rehabilitation demonstrated that low-intensity tDCS applied over the left Wernicke’s area significantly enhanced naming task accuracy and reduced response time. These results indicate that circuit-level plasticity and neuropsychological assessment scales may serve as practical and standardized functional outcome measures, suitable for integration into rehabilitation protocols during the perioperative period as well as following chemo- and radiotherapy (145). In terms of epilepsy control and patient quality of life, recent systematic reviews and real-world long-term follow-up studies have documented sustained reductions in median seizure frequency with RNS therapy, providing a methodological basis for personalized parameter optimization in brain tumor-related epilepsy (BTRE). This therapeutic approach can also be concurrently evaluated alongside AMPA receptor antagonists or gap junction uncoupling strategies to explore synergistic effects between electrical stimulation and pharmacological treatments (146).

### 6.4 Multi-target approaches: integrating anti-angiogenesis, immunotherapy, and neural circuit modulation

While anti-angiogenesis alone does not improve overall survival, it does provide symptom relief reducing edema and seizures in recurrent GBM, and perhaps opens windows for combination therapies (147, 148). There were exploratory results that were supportive of immune checkpoint inhibitors with low-dose bevacizumab, indicating the need to continue to optimize dosing schedules and identify patients (149, 150). Studies of the combination of network modulators and excitability reducers are examples of innovative multi-target combination approaches that include functional and electrophysiological endpoints, all of which are actively being studied in clinical trials. From a pharmacological delivery perspective, the implantable nine-emitter low-intensity pulsed ultrasound (LIPU) device, SonoCloud-9, has successfully completed a multicenter Phase I/II trial for recurrent glioblastoma (GBM). MRI-based quantitative assessments confirmed effective blood-brain barrier (BBB) opening under repeated ultrasound-mediated BBB disruptions combined with monthly carboplatin infusions, without encountering dose-limiting toxicities. These findings demonstrate that drug delivery coverage of broadly infiltrated tumor areas can be significantly enhanced through physical approaches. Currently, this approach has advanced to pivotal clinical trial stages, suggesting scalable clinical translation potential for strategies integrating BBB modulation with network- and microenvironment-targeting therapeutics (151, 152). Furthermore, the same platform also reported feasibility and pharmacokinetic data supporting focused ultrasound (FUS)-mediated anthracycline (doxorubicin) delivery. This establishes a temporally and spatially controllable delivery route, facilitating combinatorial treatments employing multiple therapeutic mechanisms, such as ferroptosis inducers or immunotherapeutic agents (153).

### 6.5 Locoregional CAR-T strategies aligned with peritumoral networks

Localized/intraventricular (IT/ICV) CAR-T delivery, aligned with the geometric pattern of the infiltrative tumor network, is generating emerging clinical evidence. A completed phase I clinical trial involving 65 patients receiving IL-13Rα2-targeted CAR-T therapy confirmed both feasibility and safety, with clinically meaningful disease control observed. These results strongly support spatial compatibility between CAR-T delivery and the infiltrative tumor network, particularly advantageous for multifocal and cross-network lesions (154). Additionally, the 2025 phase I trial of dual-targeted intraventricular CAR-T therapy reported measurable imaging-based lesion reduction in approximately 60% of evaluable patients, accompanied by manageable toxicity profiles. These findings provide robust support for a multi-antigen targeting strategy, coupled with sustained intraventricular CAR-T exposure, effectively addressing tumor heterogeneity and the immunosuppressive microenvironment. Concurrent communications from ASCO presentations, press releases, and formal publication in Nature Medicine mutually validated these clinical outcomes (155).

### 6.6 Bioelectrical and physical-energy therapeutics

**Figure 3:**
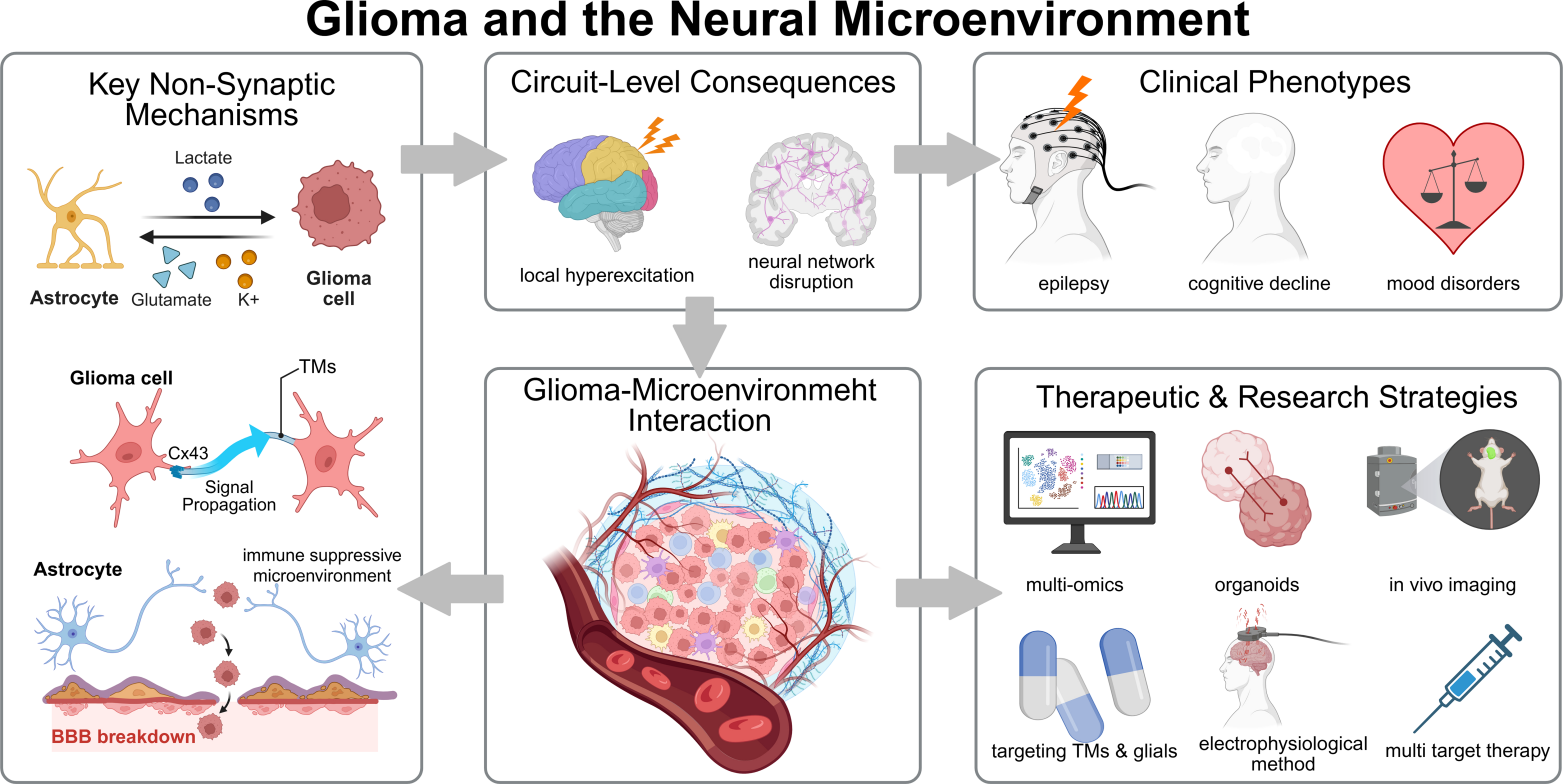
From non-synaptic mechanisms to clinical translation. Overview linking key non-synaptic pathways to circuit-level consequences and clinical phenotypes, centered on glioma–microenvironment interactions, and highlighting research/therapeutic strategies (multi-omics, organoids/brain slices, *in vivo* imaging/electrophysiology, de-networking and multi-target therapy).

Tumor treating fields (TTFields) therapy is evolving from a monotherapy that targets mitotic processes toward a broader network-disrupting modality. From a connectomic perspective, the alternating electric fields of TTFields do not merely arrest cell division; they physically perturb the structural integrity of microtubule-based extensions, specifically the tumor microtubes (TMs). By interfering with the polymerization dynamics of these macroscopic network structures, TTFields actively dismantles the physical conduits required for intercellular gap-junctional communication and calcium wave propagation, thereby undermining the collective resilience of the glioma network. Consequently, TTFields serve as an ideal network-informed foundational therapeutic platform suitable for integration with strategies targeting tumor networks and methods of BBB disruption (156, 157). See **Figure 3** for a compact guide to the strategies discussed below.

## 7 Future directions

From “synaptic interactions” to “neural network interactions”. Future glioma biology research should shift from “neuron-tumor interaction” toward the integrative framework of network neuroscience and connectomics using multi-scale modeling and graph theory to define and understand how tumors become integrated and alter the host connectome. A network neuroscience approach systematically defines interacting populations of cells, anatomic regions, and functional pathways, and encompasses all scales of organization from single cells to neural circuits. Transitioning toward this conceptual framework facilitates utilizing existing methodological and theoretical frameworks that can be directly applied to neuro-oncology in order to generate testable network-based hypotheses and intervention targets (158). From tumor elimination to reinstating neural network homeostasis. Clinical trial outcomes require alignment with this networks-focused concept. In addition to overall survival, neural circuit function and patient-reported outcomes (PROs) should be considered primary outcomes to determine whether improvement in symptoms and circuit function occurs synergistically along with survival outcomes. RANO 2.0 has provided criteria that may be used to define a consensus response outcome across a variety of treatment contexts and when there is standardization in the reporting of PRO scales which the RANO-PRO working group has thoroughly reviewed and standardized, systematic integration of symptom and functional outcomes into ongoing clinical research is feasible (72, 73). Promotion of interdisciplinary integration among Neuroscience, Oncology and Neuroengineering. The BRAIN Initiative 2.0 outlines a translational pathway from cellular and circuit systems into therapeutic intervention while outlining parallel advances and learning in the fields of technology, data science, and neuroethics. To realize this vision will involve the integration cellular and circuit dynamics with technologies for high density recording and manipulation of neuronal activity, *in-vivo* imaging, and computational neuroscience approaches all in alignment with the established methodological, theoretical, and epistemological pathways in molecular and clinical oncology. Such integration will facilitate the development of a translationally-focussed, closed-loop research paradigm that can be tracked, experimentally intervened upon, and empirically evaluated (74).

Personalization of clinical translational pathways through biomarkers and symptoms. Adaptive platform trials such as GBM AGILE and INSIGhT provide structured methods for continuous-learning, multi-arm clinical studies implemented through biomarkers and imaging- or network-based phenotypic fingerprints. The FAIR principles and BIDS ecosystem represent further developments that have laid the groundwork for multi-center capabilities that provide clinical efficacy from the PROMs and PROs through accessing, button data standards and supporting the generation evidence created through real world and multi-center resources to map network phenotypes and select appropriate interventions. Together with the radiogenomic knowledge generated from TCGA-GBM, there is potential to harness new face the imaging-to-molecular mapping to use of non-synaptic network-based metrics, neither for patient selecti0n, or as an early indication of anti-tumor treatment effectiveness (75–77).

Future studies might explore whether neuromodulation techniques aimed at restoring network excitatory-inhibitory balance can simultaneously improve clinical symptoms, such as seizures or cognitive impairment, and influence overall survival outcomes in glioma patients. Additionally, prospective validation could test whether using personalized biomarkers derived from spatial transcriptomics or connectomic data can reliably predict individual therapeutic responses and optimize intervention strategies more effectively than current standardized treatment protocols.

## 8 Conclusions

The neural microenvironment is not just a “soil” for tumors to grow. The neural microenvironment actively remodels multi-scale networks through extrasynaptic phenomena, including metabolic coupling, ionic and volume transmission, and gap-junction influenced communication, and these changes are intimately linked with tumor growth, invasion, drug resistance, and symptoms. Research in “cancer neuroscience” has firmly established the importance of these cross-scale interrelationships in tumor behavior and the biology of gliomas (155). The identified bidirectional interactions between neurons and glia lead us to conclude that neural networks are the fundamental analytical unit for the understanding of tumor behavior and clinical heterogeneity, not individual cellular units (156).

We would therefore like to propose that advancing research and clinical translation using a cellular-to-network-to-symptom-to-intervention continuum is warranted. Researchers should develop quantifiable network phenotypes, with a focus on the convergence of spatial, single-cell, and *in vivo* electrophysiological data; adopt these network phenotypes as co-primary endpoints in clinical trials; and utilize multi-arm adaptive trial designs to acheive this goal. Therapeutic translation will require novel combinations and individual-based approaches to address the distinct challenges of brain-specific immunosuppression and the physiological barriers for clinically meaningful outcomes and external validity assessments (157, 158).
